# Effects of long-term head-down-tilt bed rest and different training regimes on the coagulation system of healthy men

**DOI:** 10.1002/phy2.135

**Published:** 2013-10-31

**Authors:** Thomas Haider, Hanns-Christian Gunga, Raffaella Matteucci-Gothe, Elke Sottara, Andrea Griesmacher, Daniel L Belavý, Dieter Felsenberg, Andreas Werner, Wolfgang Schobersberger

**Affiliations:** 1 Institute for Sports Medicine Alpine, Medicine and Health Tourism UMITHall, Austria; 2 Institute of Veterinary Physiology and Zurich Center for Integrative Human Physiology (ZIHP), University of ZurichZurich, Switzerland; 3 Department for Physiology and Centre for Space Medicine, Charité University MedicineBerlin, Germany; 4 Institute for Public Health, Medical Decision Making and HTA, UMITHall, Austria; 5 Central Institute for Med. and Chem. Laboratory Diagnostics, TILAKInnsbruck, Austria; 6 Centre for Muscle and Bone Research, Charité University MedicineBerlin, Germany

**Keywords:** Hemostasis, head-down tilt, immobilization, resistance training, thromboelastography

## Abstract

Immobility plus preexisting chronic disease or acute trauma can activate the coagulation system, thus increasing the risk for thromboembolic events. The effects of long-term bed-rest immobility and microgravity on the coagulation system of healthy persons (e.g., during crewed Mars missions) have not yet been studied. The main objective of the second Berlin BedRest Study (BBR2-2) “Coagulation Part” was to investigate adaptations of the hemostatic system during long-term bed rest (60 days) under simulated microgravity (6° head-down-tilt [6°HDT]) and after mobilization in three different volunteer groups (randomly assigned to CTR= inactive control group; RE= resistive exercise only group; and RVE= resistive exercise with whole-body vibration group). In 24 males (aged 21–45 years), before, during, and after long-term bed rest, key parameters of coagulation were measured from venous blood samples: D-dimer (DD), thrombin–antithrombin III complex (TAT), and prothrombin fragment F1 + 2 (PT-F1 + 2). Additionally, modified rotational thrombelastometry (ROTEM^*®*^) analysis was performed. Times of exploratory analyses were as follows: baseline data collection 2 days before bed rest (BDC-2); eight different days of 6°HDT bed rest (HDT1–HDT60), and two different days after reambulation (R + 3 and R + 6). We found significant changes in DD, TAT, and PT-F1 + 2 over the total time course, but no consistent effect of physical interventions (RE, RVE) on these parameters. Notably, no parameter reached levels indicative of intravascular thrombin formation. All ROTEM® parameters remained within the normal range and no pathological traces were found. Sixty days of 6°HDT bed rest are not associated with pronounced activation of the coagulation system indicative of intravascular thrombus formation in healthy volunteers independent of the training type during the bed rest.

## Introduction

Immobility is a central issue in daily life and has a variety of different physiological and clinical aspects (Kahn et al. 2012). One aspect of immobility is the so-called seated immobility during long-haul travel by aircraft, car, bus, or train (Schobersberger et al. 2009). Fluid shifts with leg edema and the focus on travel-related coagulation changes are key scientific topics. Another aspect of immobility is in-hospital immobility of patients on bed rest. Moreover, a new field of immobility involves the specific situation of microgravity during spaceflights. Because research under real conditions of spaceflights is of limited possibility, head-down-tilt bed rest (HDTBR) has proven a reliable simulation model for most physiological effects of spaceflights (Pavy-Le Traon et al. 2007; Belavy et al. 2011). One adverse effect of prolonged bed rest in patients is the potential risk for the development of venous thrombosis and embolism, both of which depend on preexisting disease and accompanying treatments. Since the introduction of heparins and other anticoagulants, the incidence of in-hospital thromboembolism has decreased significantly (Kahn et al. 2009). However, these clinical findings cannot be easily transferred to HDTBR because these studies were performed in healthy volunteers. Several studies have investigated vascular adaptations but not coagulation changes after prolonged immobility and have reported an inward remodeling of conduit arteries, altered carotid wall thickness, venous enlargement, and increased distensibility and venous compliance in dependent veins (van Duijnhoven et al. 2008, 2010b; Kolegard et al. 2009).There is a paucity of data regarding bed-rest studies on subjects without preexisting disease. Rosenfeld et al. (1994) reported no change in coagulation parameters after 36 h of bed rest in healthy subjects, with the exception of an increase in plasminogen activator inhibitor 1, indicating activated fibrinolysis. With regard to HDTBR, no study has yet been conducted. Thus, it remains unclear whether a risk for venous thromboembolism during HDTBR actually exists. To prevent or reduce adverse effects of prolonged immobility—such as fluid shifts, changes in cardiovascular performance and baroreflex sensitivity, calcium metabolism, muscle mass and muscle strength, changes in bone density, bone stiffness, and bone architecture—countermeasures with mostly beneficial effects were introduced. Countermeasures include different physical exercise modalities: aerobic, resistive, and vibration exercise (Bleeker et al. 2005; van Duijnhoven et al. 2008, 2010b). There is much controversy and discussion about which countermeasure method is superior compared to others to maintain physiological functions under microgravity conditions. We hypothesized that long-term bed rest does not seriously modulate the hemostatic system (e.g., inducing a hyper-/hypocoagulable state) of healthy men.

The main objective of the second Berlin BedRest Study (BBR2-2) “Coagulation Part” was to investigate adaptations of the hemostatic system during long-term bed rest under simulated microgravity (6°HDTBR) and after mobilization in healthy volunteers performing different exercise programs.

## Material and Methods

The BBR2-2 was conducted at the Charité Campus Benjamin Franklin in Berlin, Germany, by the Centre for Muscle and Bone Research. This study was conducted according to the guidelines of the Declaration of Helsinki for research on human subjects 1989 and was approved by the ethical committee of the Charité Universitätsmedizin Berlin, Germany. All subjects provided informed written consent prior to participation in the study.

### Subjects and bed-rest design

Recruitment of volunteers started in July 2007 and the first six subjects began bed rest in September 2007. The final group of six volunteers completed bed rest in September 2008. Twenty-four medically and psychologically healthy males (mean age [SD], 32.6 [7.7] years) were recruited to undergo 60 days of strict 6°HDTBR for the BBR2-2 “Coagulation Part,” which was conducted in four campaigns of six subjects each in 2007 and 2008. HDTBR was used because this most likely simulates the physiological state of the cardiovascular system during weightlessness or spaceflight (Pavy-Le Traon et al. 2007). Medical screening included medical history, physical examination, laboratory parameters adapted from blood and urine samples, and microbiological screening. In addition, ultrasonography of pelvic and leg veins as well as kidneys was performed and, to exclude preexisting genetic hemostatic disorders, all subjects were screened for factor V Leiden mutation, factor II mutation (mutation 20210), and MTHFR mutation (A223V). A stress cardiogram and an echocardiogram were performed to attest a normal physical capacity of the candidates. The inclusion criteria were as follows: psychologically and medically healthy males aged 20–45 years, with a height range 155–195 cm, in possession of social insurance, available for more than 11 weeks (including 60 days of bed rest), and prepared to attend follow-up examinations up to 2 years after bed rest. The relevant exclusion criteria (reviewed in more detail in Belavy et al. 2010b) were as follows: any addiction (alcohol, drug, or medication), regular medical treatment or long-term hospital stays, smoker (>10 cigarettes per day) or unprepared to cease smoking for the duration of the study, regular intake of medication, chronic diseases, any type of metabolic or hormonal disturbance, history of any type of vessel disease or vessel surgery, cardiovascular diseases, disturbances of blood clotting, any type of muscle or bone disease, any acute or chronic bacterial or nonbacterial inflammatory disease, donation of blood more than 350 mL within the 3 months before the start of the study, any type of allergy, sleep disorders, and any type of joint disease (acute as well as chronic). Subjects were matched as roommate pairs upon psychological criteria (Belavy et al. 2010b) and randomized as pairs to three different groups (inactive control group, CTR; resistive exercise-only group, RE; or resistive exercise with whole-body vibration group, RVE). Methods used to implement the random allocation sequence (e.g., numbered containers or central telephone), clarifying whether the sequence was concealed until interventions were assigned. Subjects were enrolled into the study by the Center for Muscle and Bone Research (Charite University Berlin). Study organizers (D. B., D. F.) generated the allocation sequence (using random numbers obtained from http://www.random.org) and assigned subject to their groups. Randomization was done 2 days prior start of bed rest. During the baseline data collection (BDC) phase (from 9 days prior to bed rest to 1 day prior to bed rest) and prior to randomization of subjects into the different groups, all the subjects were familiarized with the exercise on three separate consecutive days (BDC-4, BDC-3, and BDC-2) to ensure that optimal training was achieved from the beginning of bed rest. Both intervention groups (RE and RVE) performed a special resistive muscle training program starting from day 1 of bed rest (HDT1) until day 57 (HDT57) on the Galileo Space exercise device (Novotec Medical GmbH, Pforzheim, Germany). All exercise interventions were performed on the same device and the 6°HDTBR position was maintained by the subjects during the entire training period. A more detailed description of subject recruitment and bed-rest design is reviewed in Belavy et al. (2010b).

### Resistive (vibration) exercise interventions

The exercise training was performed three times a week (2 × 3 min per each session) according to a standardized time schedule (described in detail in Belavy et al. 2010b). Each training session was supervised by a sports scientist. The RVE group performed similar resistive training (compared with the RE group), but it was augmented with simultaneous whole-body vibration at a certain frequency. The CTR group did not perform any type of exercise and served as an inactive control group. The sample size estimate of the BBR2-2 project was based on bone parameters (Belavy et al. 2010b) not those of this study. Because no preexisting data exist regarding the effects of HDTBR on hematological parameters, it is not possible to perform a sensitivity analysis to determine the required size effect for this study. Hence, we consider the current work an exploratory study for the effects of bed rest and exercise on coagulation parameters.

### Briefly, in a given exercise session, the following exercises were performed

1. *Warm up*: bilateral squat exercise (from 10° to 90° of knee flexion and back) with 50% of the maximal force for 64 sec (eight repetitions; RVE group, plus 24-Hz vibration; 2-min break thereafter).
2. *Bilateral squats* (from 10° to 90° of knee flexion and back) on submaximal levels continuously until exhaustion (gradually increased force levels to reach a maximum of eight repetitions; RVE group, plus 24-Hz vibration; 5-min break thereafter).
3. *Single-leg heel raises* on the left and right legs from maximal plantar flexion to maximal dorsiflexion against a force equivalent to ˜1.3 times the HDT1 body weight as quickly as possible until exhaustion (gradually increased force levels to reach a maximum of 30 sec per single leg; 90-sec break after each single leg; 4-min break after both legs; RVE group, plus 26-Hz vibration).
4. *Double-leg heel raises* in the same manner as for single-leg heel raises except that the resistive force was set to ˜1.8 times the body weight (gradually increased force levels to reach a maximum time interval of 40–50 sec until exhaustion; RVE group, plus 26-Hz vibration; 2-min break thereafter).
5. *Back and toe raise* with their feet positioned on the platform, subjects extended their hips and lumbar spine, dorsiflexed their ankles, and maintained their knee at full extension (constant nonprogressive resistive force of 1.5 times the body weight for 60 sec; RVE group, plus 16-Hz vibration).

### Blood sampling and analytical methods

Venous blood was collected from a cubital vein without stasis between 7 and 8 am in the morning. Erythrocyte count, hemoglobin (Hb) concentration, hematocrit (Hct), and platelet count were analyzed with standard laboratory methods (Bayer ADVIA 120 automated hematology analyzer, Siemens Diagnostics, Eschborn, Germany). The relative changes in Hct and Hb were used to estimate relative changes in plasma volume (PV) using baseline values (BDC-2) as the reference to compare with values obtained from single days of HDTBR (Dill and Costill 1974; Johansen and Norsk 1995). In addition, the following key parameters of coagulation were determined by appropriate standard laboratory tests: D-dimer (DD; Innovance D-dimer, a particle-enhanced immunoturbidimetric assay; Dade Behring, Marburg, Germany), thrombin–antithrombin III complex (TAT; Enzygnost TAT micro, Dade Behring), and prothrombin fragment F1 + 2 (PT-F1 + F2; Enzygnost F1 + 2 monoclonal, Dade Behring). Because of the limited blood volume available, no further parameter of hemostasis could be analyzed.

### The modified rotational thrombelastometry analyzer

The rotational thrombelastometry (ROTEM®, Tem Innovations GmbH, Munich, Germany) coagulation analyzer (Pentapharm, Munich, Germany) includes four independent measurement channels and is an enhancement of classical thromboelastography (TEG). The ROTEM® system uses a ball-bearing system for power transduction, making it less susceptible to mechanical stress, movement, and vibration. The ROTEM® technique and its parameters have been described in detail elsewhere (Luddington 2005; Franz 2009; Park et al. 2009; Chitlur and Lusher 2010). For additional monitoring of routine coagulation tests in subjects, the following parameters were automatically detected by the ROTEM® analysis software based on thromboelastograms: clotting time (CT), clot formation time (CFT), alpha angle (ALP), maximum clot firmness (MCF) after 15 min in EXTEM (activation of coagulation via tissue factor) and INTEM (activation of coagulation via the contact phase), and MCF after 15 min in FIBTEM (activation of coagulation via tissue factor and cytochalasin D). Although the ROTEM® system is currently mainly established and used for the control and differential diagnosis of hemostatic disorders within the context of acute bleeding, recent literature has also suggested a possible role of the ROTEM® system in testing for hypercoagulable states (Franz 2009; Park et al. 2009). For ROTEM® analysis, citrated blood samples were obtained. All ROTEM® thromboelastograms were performed by the same individual.

### Statistical analysis

**Figure 1:**
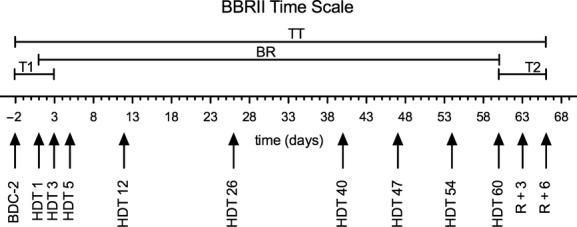
Time scale of the second Berlin BedRest Study “coagulation part”. TT, total observation period; BR, 60 days of 6° head-down-tilt bed rest (6°HDTBR), BDC-2 (2 days prior to the beginning of bed rest); *T*1, transition from the upright position to 6°HDT (BDC-2 to HDT3); *T*2, reambulation from bed rest (HDT60 to R + 6).

Measurements and analytical methods were performed at different times: baseline data collection 2 days before bed rest (BDC-2), days 1 (HDT1), 3 (HDT3), 5 (HDT5), 12 (HDT12), 26 (HDT26), 40 (HDT40), 54 (HDT54), and 60 (HDT60) of 6°HDTBR, and days 3 (R + 3) and 6 (R + 6) after reambulation. Based on the change in body position, the entire observation period (TT) (BDC-9 to R + 6) was further subdivided into the following intervals (Fig. 1): transition from the upright position to 6°HDTBR (*T*1, BDC-2 to HDT3), bed rest (BR, HDT1 to HDT60), and reambulation from bed rest (T2, HDT60 to R + 6).

Data are presented either as mean values and SD, or as mean values and SEM (Fig. 4). A nonparametric analysis of variance type (box approximation for small sample sizes with chi-squared degrees of freedom; *B*-value) according to Brunner and Langer (1999) was used for testing main effects on hematological parameters, relative changes in plasma volume (PVC), parameters of hemostasis and fibrinolysis (Table 1), and ROTEM® parameters (Table 3) across the TT as well as across subdivided time intervals (*T*1, BR, T2). Main effects have been statistically tested for “time,” “group,” and the interaction of both factors. Nonparametric statistics were applied for comparisons at BDC-2: the Kruskal–Wallis test for comparison across the three groups, the Mann–Whitney *U*-test for pairwise comparison, and the Wilcoxon signed-rank test for paired testing between two times. A two-tailed *P*-value less than 0.05 was deemed to be statistically significant. All statistical calculations were performed using commercially available statistical software (SAS, vers. 9.1; SPSS vers. 19.0; Chicago, IL).

**Table 1:** Longitudinal analysis of hematological parameters and parameters of hemostasis and fibrinolysis

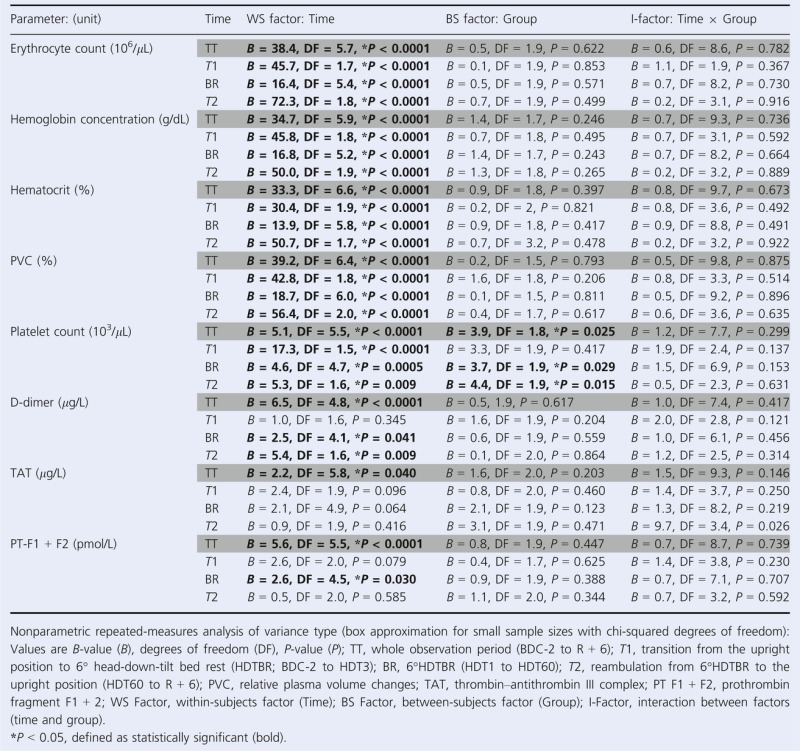

**Table 2:** ROTEM® parameters at selected times

		Time							
	
Parameter (unit)		BDC-2	HDT1	HDT3	HDT12	HDT26	HDT60	R + 3	R + 6
EXTEM									
CTR	CT (sec)	54 (48;55)	62 (55;67)	54 (50;64)	56 (47;58)	54 (50;57)	64 (54;73)	52 (46;62)	59 (52;64)
	CFT (sec)	103 (92;109)	101 (89;110)	95 (85;105)	94 (86;103)	93 (86;105)	89 (77;95)	80 (75;86)	75 (67;86)
	ALP (°)	70 (69;72)	70 (69;72)	71 (69;73)	71 (70;73)	71 (70;73)	72 (71;75)	74 (73;75)	75 (73;76)
	MCF (mm)	58 (56;62)	60 (55;61)	61 (55;62)	62 (56;64)	60 (57;61)	61 (58;63)	64 (59;65)	65 (59;68)
RE	CT (sec)	56 (51;63)	56 (53;58)	55 (51;62)	54 (52;60)	52 (46;56)	59 (54;59)	54 (51;56)	55 (52;60)
	CFT (sec)	103 (98;125)	99 (82;120)	92 (87;98)	90 (82;101)	100 (83;111)	99 (81;112)	88 (83;101)	90 (83;114)
	ALP (°)	70 (65;71)	72 (67;74)	72 (70;72)	72 (71;74)	70 (68;74)	70 (68;74)	73 (70;74)	72 (67;74)
	MCF (mm)	58 (51;61)	58 (56;60)	62 (59;63)	61 (60;62)	59 (53;60)	59 (55;63)	62 (56;64)	60 (54;64)
RVE	CT (sec)	58* (56;62)	59 (55;62)	61 (51;63)	57 (55;58)	60 (51;62)	53 (50;57)	60 (52;68)	55 (52;56)
	CFT (s)	97 (82;157)	93 (72;128)	83 (79;121)	82 (73;89)	89 (83;111)	84 (78;101)	78 (67;107)	79 (70;92)
	ALP (°)	70 (60;74)	71 (65;75)	73 (66;74)	74 (73;75)	72 (68;74)	73 (70;74)	74 (69;76)	75 (72;76)
	MCF (mm)	58 (48;62)	59 (51;62)	59 (54;64)	63 (60;65)	59 (56;64)	59 (57;64)	61 (58;65)	65 (59;67)
FIBTEM									
CTR	MCF (mm)	12 (11;13)	12 (10;14)	13 (11;16)	13 (12;16)	13 (12;15)	14 (13;15)	17 (14;18)	16 (14;21)
RE	MCF (mm)	14 (11;16)	12 (10;14)	14 (12;15)	14 (14;15)	14 (10;15)	13 (9;17)	15 (13;17)	15 (11;17)
RVE	MCF (mm)	13 (11;16)	12 (11;14)	13 (11;17)	15 (13;18)	13 (10;16)	12 (11;17)	14 (12;18)	15 (13;17)

Median values (Q1; Q3) of ROTEM® parameters (CT, CFT, ALP, MCF) for EXTEM tests, and MCF for FIBTEM in different groups (CTR, RE, RVE) at selected times: Baseline (BDC-2), bed-rest (HDT1–HDT60), and after reambulation (R + 3, R + 6); Comparison between groups at selected times, MW-*U-*test:

* *P* < 0.025 vs. CTR.

**Table 3:** Longitudinal analysis of ROTEM® parameters

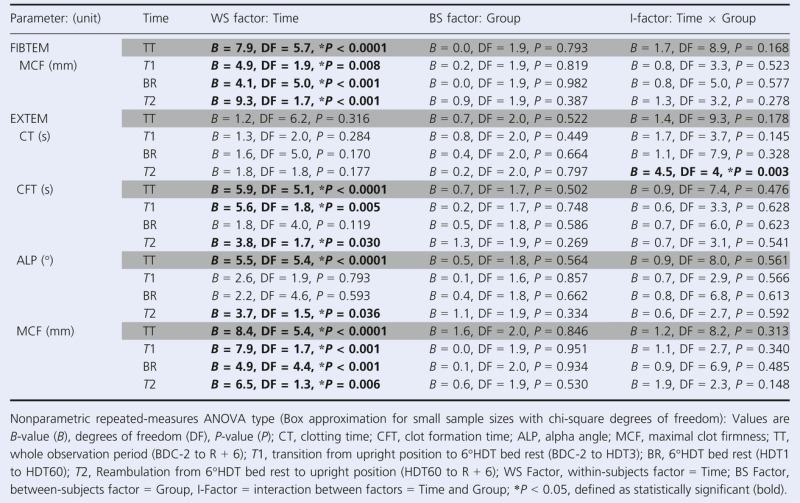

**Figure 2:**
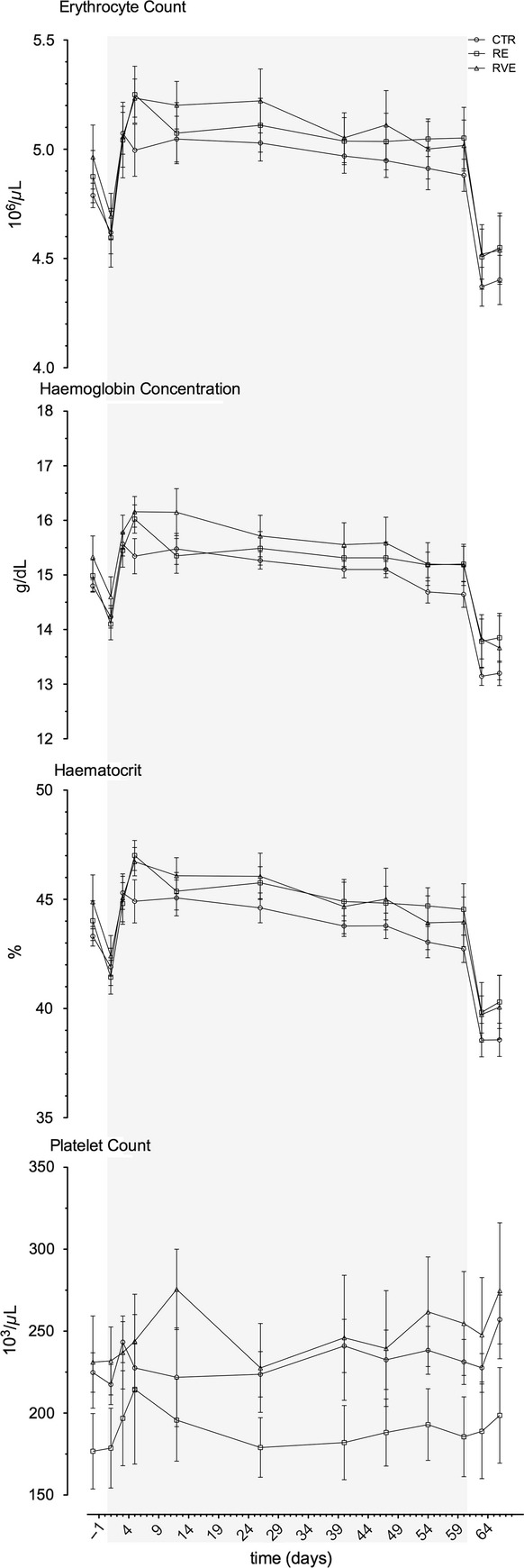
Longitudinal observation of selected blood parameters starting at BDC-2 (day 2 prior to the beginning of bed rest) across 60 days of 6°HDTBR (days 1–60; horizontal bar) to days 3 and 6 post–bed rest (days 63 and 66). CTR, inactive control group; RE, resistive exercise-only group; RVE, resistive exercise with whole-body vibration group. Data are expressed as means (error bars, standard error of the mean [SEM]). Gray area indicates the period of bed rest.

**Figure 3:**
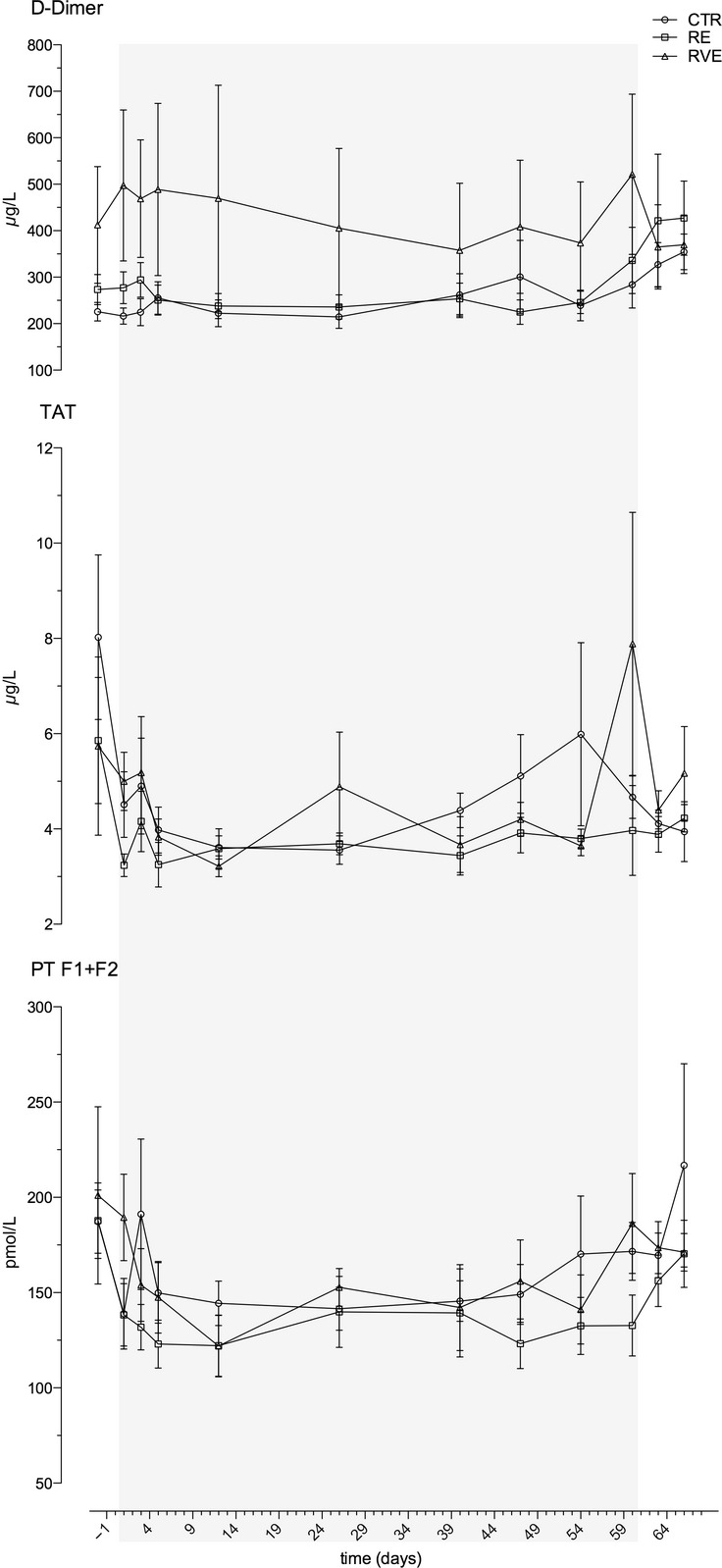
Longitudinal observation of hemostasis starting at BDC-2 (day 2 prior to the beginning of bed rest) across 60 days of 6°HDTBR (days 1–60; horizontal bar) to days 3 and 6 post–bed rest (days 63 and 66). CTR, inactive control group; RE, resistive exercise-only group; RVE, resistive exercise with whole-body vibration group. TAT, thrombin–antithrombin III complex; PT F1 + F2, prothrombin fragment F1 + 2. Data are expressed as means (error bars, standard error of the mean [SEM]). The gray area indicates the period of bed rest.

**Figure 4:**
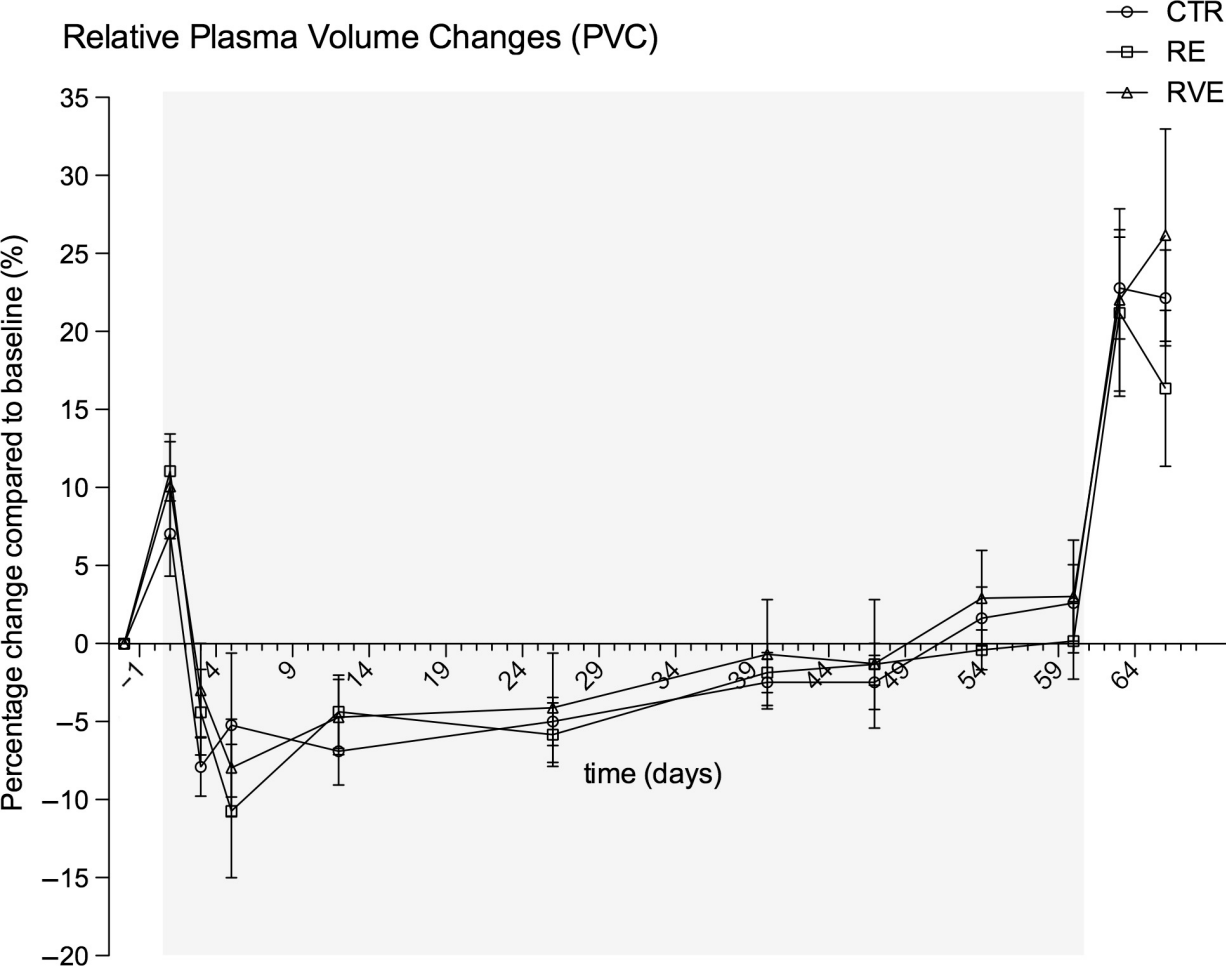
Longitudinal observation of relative changes in plasma volume (PVC) estimated via relative changes in hemoglobin and hematocrit values with reference to baseline values (BDC-2) across 60 days of 6° head-down-tilt bed rest (HDTBR; days 1–60; horizontal bar) to days 3 and 6 post–bed rest (days 63 and 66). CTR, inactive control group; RE, resistive exercise-only group; RVE, resistive exercise with whole-body vibration group. Data are expressed as means (error bars, standard error of the mean [SEM])]. The gray area indicates the period of bed rest.

## Results

At the beginning of bed rest, there was no difference (*F*, all <1.2; *P* > 0.33) among groups (CTR, *n* = 9; RE, *n* = 8; RVE, *n* = 7) regarding age (group, mean ± SD, unit: CTR, 33.1 ± 7.8 years; RE, 31.1 ± 5.1 years; RVE, 32.2 ± 10.4 years), body weight (CTR, 80.6 ± 5.2 kg; RE, 75.0 ± 12.8 kg; RVE, 81.5 ± 6.2), and height (CTR, 181.3 ± 6.0 cm; RE, 179.3 ± 7.7 cm; RVE, 179.6 ±5.8 cm). Twenty-three subjects successfully completed 60 days of 6°HDTBR. One subject (RE group) left bed rest on day 30 of bed rest (HDT30) for medical reasons unrelated to this study. Another subject of the RVE group had already at baseline an abnormal high DD value, whereas the rest of the subject's hemostatic parameters were within the normal reference range and ultrasonography of leg and pelvic veins did not show any evidence of a deep venous thrombosis. The subject was not excluded from the data analysis as a solely high DD level was not defined as exclusion criteria. No severe, serious, or unexpected adverse events were observed due to bed rest or resistive exercise interventions. None of the measured key parameters for coagulation reached levels was indicative of intravascular thrombin formation. All ROTEM® parameters remained within the normal range.

### Blood parameters, PVC, and markers of coagulation and fibrinolysis

At BDC-2, there was no significant difference among groups regarding blood parameters such as erythrocyte count, Hb concentration, and Hct with the exception of the thrombocyte count, which was significantly lower in the RE group compared with the CTR group (mean ± sd, 177 ± 28 × 10^3^/μL vs. 225 ± 36 × 10^3^/μL, respectively; *P* = 0.016; Fig. 2). There was no significant difference at BDC-2 among groups for the parameters of coagulation and fibrinolysis, such as DD, TAT, and PT-F1 + F1 (Fig. 3). There was also no significant difference after 60 days of bed rest (HDT60) compared with the baseline (BDC-2) for both hematological parameters and parameters of coagulation and fibrinolysis. Longitudinal analysis (Table 1) showed a significant time effect for hematological parameters such as erythrocyte count, Hb concentration, and Hct across the TT and also across defined time intervals (*T*1, BR, T2) for all groups but not among groups and not for the interaction between the factors time and group. The three hematological parameters followed the same pattern along the observation period when compared to baseline levels (Fig. 2). At the beginning of bed rest (HDT1) the values initially decreased, followed by an increase within the next couple of days (HDT3 to HDT5) peaking at HDT5, and dropping slightly till the end of bed rest (HDT60) with another decrease after reambulation peaking at R + 3. Because the relative changes in hematological parameters (Hb concentration and Hct) were used to estimate the relative plasma volume changes, PVC confirmed the significant time effect across all time segments (TT, *T*1, BR, *T*2) for all groups without showing any effects among groups or for the interaction between the factors time and group (Table 1 and Fig. 4). The PVC showed an initial increase after changing the body position from upright to 6°HDTBR at HDT1 in all groups (group mean ± SD, +9.3 ± 7.5%), followed by a decrease at HDT3 (−5.3 ± 7.3%), and again reaching comparable values (slightly increased mean, +1.9 ± 7.6%) to baseline (BDC-2) after 60 days of HDTBR. A stronger relative increase in PV (+21.8 ± 12.1%) was observed at day 3 after the reambulation from HDTBR (R + 3). In contrast, the changes in platelet count showed a less clear pattern along the observation period (Fig. 3). The longitudinal analysis for the platelet count showed only a significant time effect for TT, *T*1, and *T*2 but not for BR. Additionally, a significant group effect was observed between CTR and RE for TT (*B* = 8.8; DF = 1.0; *P* = 0.003), BR (*B* = 8.1; DF = 1.0; *P* = 0.004), and *T*2 (*B* = 9.3; DF = 1.0; *P* = 0.002). There was a significant time effect for DD across TT, BR, and *T*2 but not across *T*1, whereas TAT showed only a significant time effect across TT, and PT-F1 + F2 showed a significant time effect across TT and BR. None of the parameters of hemostasis and fibrinolysis showed a significant difference among groups or a significant interaction between the factors time and group.

### ROTEM® parameters

At BDC-2, there was no significant difference among groups for all measured ROTEM® parameters (Table 2). Notably, in the comparison between the baseline (BDC-2) and after 60 days of bed rest only, the CTR group showed in the FIBTEM analysis significantly increased MCF (mean ± sd, 12 ± 2 mm vs. 14 ± 3 mm, respectively; *P* = 0.036) and, in the EXTEM analysis, a decreased CFT (101 ± 12 sec vs. 86 ± 12 sec, respectively; *P* = 0.038) and an increased ALP (70 ± 3° vs. 73 ± 2°, respectively; *P* = 0.049), whereas the training groups (RE and RVE) showed no significant difference for any ROTEM® parameter in the pre–post comparison. Longitudinal analysis (Table 3) exhibited a significant time effect in the FIBTEM analysis for MCF across TT and also across all subdivided time intervals (*T*1, BR, *T*2). In addition, there was a significant interaction between factors time and group across TT. In the INTEM analysis (data not shown), there was a significant time effect for ALP and MCF across TT and subdivided time intervals (*T*1, BR, *T*2) with the exception of CFT, which showed no significant time effect across TT and *T*1. No significant effect at any time was observed for CT. The EXTEM analysis showed a significant time effect across TT for CFT, ALP, and MCF. In the subsegment analysis, there was only a significant time effect for CFT and MCF across *T*1 and for CFT, ALP, and MCF across *T*2 but not across BR. The CT showed again no significant time effect at any time. In the entire ROTEM® analysis, there were only two exceptions showing a significant I-Factor, one within FIBTEM analysis (MCF across TT) and one within the EXTEM analysis (CT across *T*2). In general, no consistent group effect or consistent interaction of factors time and group was observed for the evaluated ROTEM® parameters. In addition, all ROTEM® parameters remained within the normal reference ranges.

## Discussion

The aim of the BBR2-2 study (“coagulation part”) was to investigate the consequences of 60 days of prolonged bed rest (6°HDT) on parameters of coagulation and fibrinolysis. Furthermore, one study goal was to investigate the possible influence of different training modalities during bed rest (i.e., resistive exercise and resistive exercise plus whole-body vibration) on the hemostatic system. To our knowledge, BBR2-2 is the first bed-rest study with that focussed mainly on hemostasis. As key findings, we measured significant changes in DD, TAT, and PF-F1 + 2 over the total time course of the study. However, none of these parameters reached plasma concentrations indicative of intravascular thrombus formation. Some of the ROTEM® parameters showed significant changes over time; however, the absolute values remained within the reference ranges and no pathological trace was found. None of the measured parameters was influenced by the applied countermeasures (no consistent group effects or interaction of time and group).

### Bed rest and hemostasis control group

Over the TT, time effects for DD, TAT, and PF-*T*1 + 2, as well as for ROTEM® parameters were detected. To also detect possible time effects for shorter periods, we clustered the total study period into the phase from the upright position into the 6°HDTBR position (*T*1), into the bed-rest phase (BR), and into the reambulation period (*T*2). Moreover, data were analyzed for selected days during the entire study. For the control group, there were some fluctuations among the different days. However, overall, there was no evidence for an activation of coagulation or fibrinolysis. In the ROTEM® analysis, a “hypercoagulable” trace shows a shortened CT, an increased ALP, and an increased MCF. Vice versa a “hypocoagulable” state shows a prolonged CT, a decreased ALP, and a decreased MCF (Luddington 2005). Although we found significant changes in several ROTEM® parameters over the total time course, we did not find ROTEM® traces, which were clearly indicative for a hyper- or hypocoagulable state. Thus, the ROTEM® results further support the main hypothesis that long-term bed rest is not associated with profound changes in the hemostatic system.

### Effects of countermeasure exercises

An essential part of the entire BBR2-2 project was the evaluation of different countermeasure exercises to combat the negative consequences of human spaceflight on the musculoskeletal and vascular systems (Belavy et al. 2010b). Therefore, two exercise modalities were selected: (1) resistive exercise alone (RE) or (2) resistive exercise in combination with whole-body vibration (RVE). Some of the already published main BBR2-2 results are as follows: High-load resistive exercise countermeasures (with and without vibration) incorporating squat exercises reduced and/or prevented atrophy of the posterolateral hip and hamstring musculature. Addition of whole-body vibration had no supplementary protective effect (Miokovic et al. 2011). Regarding bone loss after 60 days of bed rest, the combination of whole-body vibration and high-load resistive exercise was more efficient than RE alone (Belavy et al. 2011). RE without or in combination with whole-body vibration could reduce lumbar muscle atrophy (Belavy et al. 2010a). Bed rest induced a rapid increase in wall thickness of the carotid and femoral arteries in the participants, a finding that was counteracted either completely (carotid artery) or largely (superficial femoral artery) by exercise countermeasures (van Duijnhoven et al. 2010a). RVE, but not RE, could abolish the marked increase in flow-mediated dilation in the superficial femoral artery after 60 days of bed rest and could reduce the baseline diameter and dilator capacity, which is a regular phenomenon with prolonged bed rest (van Duijnhoven et al. 2010b). However, all other authors from the BBR2-2 study concluded that further countermeasure optimization is required. Whole-body vibration was recently shown to augment fibrinolytic activity in healthy men when combined with squatting exercises (Boyle and Nagelkirk 2010). Therefore, one could speculate that RVE during bed rest may also activate fibrinolysis. Regarding countermeasure exercise and hemostasis, there was obviously no influence of RVE or RE on coagulation and fibrinolysis because DD, TAT, and PF-F1 + 2, as well as all ROTEM® parameters, showed a similar time course among the three groups tested. Even splitting the TT into shorter phases (*T*1, BR, and *T*2) did not reveal different group effects or significant interactions between the factors time and group. As the effects of bed rest on coagulation and fibrinolysis per se were small in magnitude in the CTR group and not clinically relevant, there is probably less need for (exercise) countermeasure protocols to prevent these changes. However, effective countermeasure protocols are of great importance to maintain functional homeostasis in other physiological systems (e.g., the musculoskeletal system).

### Bed rest and relative plasma volume changes

Independent of the group contribution, there were significant PVC in the early phase of bed rest (*T*1) and early after reambulation (*T*2). The transient initial increase in PV of ∼10% between BDC-2 and HDT1 was followed by a decrease of ∼5% at HDT3, and comparable values to baseline were estimated after 60 days of HDTBR (HDT 60). At day 3 after reambulation from HDTBR (R + 3), an increase of ∼20% was estimated compared with BDC-2. Such volume (fluid) shifts due to changes in body position have been well known and have been proposed as true changes in plasma volume (reviewed in Pavy-Le Traon et al. 2007). Changes in Hb and Hct were used to estimate the changes in PV during bed rest (Dill and Costill 1974; Greenleaf 1984; Johansen et al. 1992; Johansen and Norsk 1995), and the estimated changes in PV had been compared to reliable methods to measure PVC such as the Evans blue (EB) dilution technique (Foldager and Blomqvist 1991; Johansen et al. 1997). However, it was also postulated that, particularly in the early phase of postural changes, the Hb–Hct method seemed to underestimate PVC at least when compared with EB (Johansen et al. 1998). Based on the limitation of the Hb–Hct method during the early phase of postural changes and our data, which suggested that the PVC during prolonged HDTBR seemed to be small in all groups with a clear tendency toward baseline values at the late phase of HDTBR, we did not correct the blood parameters for PVC. Furthermore, the estimated PVC data showed a consistent time effect in all groups for the tested time intervals (TT, *T*1, BR, and *T*2) but no group effect or any interaction. Depending on the duration of bed rest or spaceflights and the methods used for PVC analysis, the absolute changes reported were decreases between 10% and 15%, which were quite similar between bed rest and spaceflights (Fortney et al. 1991; Johansen et al. 1997; Pavy-Le Traon et al. 2007). The main reason for this significant PV reduction is the upward shift in the thoracocephalic fluid volume with the transient increase in PV stimulating central volume carotid, aortic, and cardiac receptors that induce accelerated diuresis and natriuresis. During BBR2-2, fluid intake and urine output both increased significantly without any difference among the three groups (Belavy et al. 2010b). After reambulation (R + 3, R + 6), Hct and hemoglobin levels decreased; thus, the calculated plasma volume expanded. In addition, slow reductions in red blood cell volume were reported after several weeks of bed rest (Fortney et al. 1991). Because we did not measure red blood cell volume, the contribution of a real reduction in red blood cell volume during bed rest and after reambulation remains unclear. Remarkably, in our BBR2-2 study, the PV shifts were independent of the countermeasure exercises performed.

### Comparison of BBR2-2 with other studies on immobility

One well-known situation regarding immobility is the so-called seated immobility during long-distance travel by air, bus, train, or car where travelers must assume a cramped position for several hours, thus worsening leg vein flow conditions. In our previous studies, we could demonstrate a certain activation of coagulation during long-haul flights (Schobersberger et al. 2002) and long-distance bus travel (Schobersberger et al. 2004). TEG measurements revealed that moderate activation of coagulation and PT-F1 + 2 was elevated after travel by aircraft and bus. TAT and DD remained unchanged under both conditions. Moreover, after air travel, suppression of fibrinolysis was measured. However, bed rest and long-distance travel are quite different situations of immobility and making a comparison regarding their data is difficult. Aboard the International Space Station, exercise training of 1.5–2 h of treadmill training is performed on a daily basis by the astronauts. However, with the results of this study, the effects of long-term endurance exercise-induced coagulation changes cannot be predicted during prolonged flights.

### Limitations of the present study

Because coagulation measurements represented one part of the entire BBR2-2 project, the relatively low number of participants for an intervention study had to be accepted. Thus, some findings of no observed differences may represent false negatives. Moreover, there was a definite restricted blood volume available at each time point for hemostasis, a finding that limited the analyzed number of different parameters of coagulation and fibrinolysis. From a logistical point of view, it was not possible to collect blood before day 3 (R + 3) after reambulation. Thus, possible early effects of reambulation could not have been detected. Although most of the observed changes in hematological parameters and parameters of hemostasis due to postural changes seemed to already peak at day R + 3, we currently do not exactly know the time when all investigated parameters had returned to baseline levels.

## Conclusions

Despite the extraordinary immobility period of 60 days bed rest, there was no evidence of clinically relevant changes in the hemostatic system indicative of either an activation of coagulation and/or a suppression of fibrinolysis. No training intervention could influence coagulation and fibrinolysis. Regarding space medicine, our study indicates for the first time that, at least in healthy subjects, hemostasis may not be a critical factor for prolonged space missions.
